# Is There Need for a New Hepatitıs B Vaccine Schedule for Children with Celiac Disease?

**DOI:** 10.5812/kowsar.1735143X.715

**Published:** 2011-08-01

**Authors:** Vildan Ertekin, Mahya Sultan Tosun, Mukadder Ayse Selimoglu

**Affiliations:** 1 Department of Pediatric Gastroenterology, Hepatology and Nutrition, Istanbul Faculty of Medical Sciences, Istanbul University, Istanbul, Turkey; 2 Department of Pediatric Gastroenterology, Hepatology and Nutrition, Faculty of Medical Sciences, Ataturk University, Erzurum, Turkey; 3 Department of Pediatric Gastroenterology, Hepatology and Nutrition, Faculty of Medical Sciences, Inonu University, Malatya, Turkey

**Keywords:** Celiac disease, Hepatitis B vaccine, Child

## Abstract

**Background:**

Celiac disease (CD) is an autoimmune disease characterized by immunemediated inflammatory damage of the small intestinal mucosa, precipitated by the ingestion of gluten-containing foods. Since human leucocyte antigen DQ2 (HLA-DQ2) is a marker of nonresponsiveness to hepatits B virus (HBV) vaccine, CD may also be associated with this nonresponsiveness.

**Objectives:**

The aim of this study was to compare the responses to HBV vaccine between children with CD and healthy children. We also investigated the relationship between the patients’ responses to hepatitis B vaccine, the clinical presentation of CD, and dietary compliance in the patients.

**Patients and Methods:**

We recruited 52 children with CD and 20 age- and sex-matched healthy children who received HBV vaccination according to the standard immunization schedule. The production of specific antihepatitis B surface antigen (HBsAg) antibodies was evaluated in all patients and control participants. Subjects with less than 10 IU/L anti-HBs were consided nonresponders to the vaccination.

**Results:**

31 of the 52 patients (59.6%) were female and 21 (40.4%) were male. The mean age of the CD patients was 10.7 ± 4 years (range, 4–18 years). Anti-HBs titers were positive in 32 (61.5%) patients and negative in 20 (38.5%) patients, while they were positive in 18 (90%) of the children in the control group (P < 0.05). We found statistically significant differences between negative anti-HBs titers, clinical presentation of CD, and dietary compliance in patients with CD (P < 0.05).

**Conclusions:**

Nonresponsiveness to hepatitis B vaccination was more frequently found in children with CD than in the control group. Therefore, the response to HBV vaccination should be investigated in children with CD, and a different immunization schedule may need to be developed. Further, compliance to the prescribed gluten-free diet (GFD) may improve the immune response to HBV vaccination in children with CD.

## 1. Background

CD is a lifelong, gluten-sensitive, intestinal enteropathy with multifactorial etiology. The disease provides an exciting model where both genetic andenvironmental factors play an important role [1]. The estimated prevalence of CD in the general population of North America and Western Europe is close to 1% [2]. The first study on CD prevalence in Turkey reported that the prevalence was 1:115 [3]. Genetic, environmental, and immunological factors may play a role in the pathogenesis of the disease. CD is associated with the extended major histocompatibility complex (MHC) haplotypes B8, DR3, and DQ2, but most specifically with DQ2. In fact, human leucocyte antigen (HLA) DQ2 is found in 90–95% of celiac patients, and individuals lacking the DQ2 haplotype are generally positive for  DQ8 [4][5][6]. Rostami Nejad et al. found that weight loss and constipation appeared to be correlated significantly with CD. This atypical presentation, especially the correlation with constipation, has received considerably less attention in clinical practice than typical forms of disease such as diarrhea and malabsorption [7]. HBV infection is an important global public health problem;one-third of the world’s population has been infected with HBV, which has caused acute and chronic liver disease, cirrhosis, and hepatocellular carcinoma [4][5][6][8][9]. Hepatitis B vaccines were introduced in the early 1980s [10]. The implementation of mass immunization programs, which have been recommended by the World Health Organization since 1991, has dramatically decreased the incidence of HBV infection among infants, children, and adolescents in many countries [5]. In our country, a routine immunization program was initiated by the Ministry of Health in 1998 [9]. Protective serum titers of anti-HBs (greater than 10 IU/L) develop in 95–99% of healthy infants, children, and young adults who receive a series of 3 intramuscular doses [10]. In the healthy population, 4–10% of vaccine recipients fail to produce protective levels of antibodies to the HBV after standard immunization depending on age and the presence of various underlying diseases [4][8][11]. Many nongenetic factors including age, obesity, smokin, drug abuse, alcoholism, infections, immunosupression, and route of vaccination are known to be associated with nonresponsiveness [8]. Nonresponders are often found to carry specific HLA haplotypes including B8, DR3, and DQ2 [6]. CD may be associated with nonresponsiveness to the HBV vaccine since HLA genotypes play an important role in nonresponsiveness.

## 2. Objectives

The aim of this study was to compare the responses to HBV vaccination between children with CD and healthy children. We also investigated the relationship between the response to the HBV vaccine, clinical presentation of CD, and dietary compliance in celiac patients.

## 3. Patients and Methods

A total of 52 patients who were diagnosed with CD and received the HBV vaccine according to the standard immunization schedule in our pediatric gastroenterology department were included in the study. The inclusion criteria required that subjects must have completed HBV vaccination at least 6 months before enrolment in this study. Patients who had received any drug treatments were not enrolled in the study. The diagnosis of CD was based on the criteria outlined by the European Society for Paediatric Gastroenterology, Hepatology, and Nutrition (ESPGHAN) [12]. Biopsies of the small intestine were performed for all patients, and all biopsy specimens were evaluated according to the modified Marsh criteria [13]. Atypical presentation of CD included symptoms such as constipation, dyspepsia, abnormal results of liver function tests, and alopecia areata, while clinical presentation of classical CD included symptoms of malabsorption and failure to thrive. The control group was composed of 20 age- and sex-matched healtychildren. Recombinant hepatitis B vaccine (Engerix B, Glaxo Smith and Kline, Belgium) was administered to all study participants, including CD patients and healthy controls. All CD patients and control subjects were examined to determine the presence of HBsAg and HBV core antibody immunoglobulin G (anti-HBcIgG), and the quantity of anti-HBs. These HBV markers were measured by standard enzymelinked immunosorbent assay (ELISA; Organon Teknika Hepanostika, Holland). Subjects with anti-HBs less than 10 IU/L were considered nonresponders to the vaccine. HBsAg and anti-HBcIgG were negative in all CD patients and in the control group. Informed consent was obtained from the parents of each child. The study was approved by the Ethical Committee of Ataturk University. Statistical analyses were carried out using SPSS 12.0.

## 4. Results

31 of the 52 patients (59.6%) were female, and 21 (40.4%) were male. The mean age of the patients with CD was 10.7 ± 4 years (range, 4–18 years). 38 (73.1%) of the patients with CD had typical CD presentation, while 14 (26.9%) patients had atypical CD presentation. Among the CD patients, 10 (19.2%) had another accompanying disease (insulin-dependent diabetes mellitus, 4 [7.6%] patients; autoimmune hepatitis, 3 [5.8%] patients; or epilepsy, 3 [5.8%] patients). 44 (84.6%) CD patients were compliant with the prescribed gluten free diet (GFD), whereas 8 (15.4%) were noncompliant. Anti-HBs titers of CD patients were found to be positive in 32 (61.5%) patients and negative in 20 (38.5%) patients, while 90% (18/20) of control participants had positive anti-HBs titers. The anti-HBs positivity in CD patients was significantly lower than that of the control participants (P < 0.05). The relationships between anti-HBs titers and sex, presentation, coexisting disease, and dietary compliance in CD patients is summarized in Table.

**Table:** The Relationship between Anti-HBs Titers and Sex, CD Presentation, Coexisting Diseases, and Dietary Compliance in CD Patients

	**Anti-HBs**		**P value**
	**Negative, No. (%)**	**Positive, No. (%)**	
Sex			0.0001
Female	4 (12.9)	27 (87.1)	
Male	16 (76.2)	5 (23.8)	
Presentation			0.008
Typical	19 (50)	19 (50)	
Atypical	1 (7.1)	13 (92.2)	
Coexisting disease			0.4
Yes	5 (50)	5 (50)	
No	15 (35.7)	27 (64.3)	
Dietary compliance			0.04
Yes (tTG IgA a: negative)	14 (31.8)	30 (68.2)	
No (tTG IgA a: positive)	6 (75)	2 (25)	

^a^ Abbreviation: tTG IgA, Tissue transglutaminase immunoglobulin A

## 5. Discussion

Celiac disease, the most common food-sensitive enteropathy in humans, is an autoimmune disease affecting primarily the proximal small intestine because of a permanent intolerance for dietary gluten [14][15]. Genetic, environmental, and immunological factors may all play a role in the pathogenesis of CD [3][4][5]. CD can occur together with a number of endocrinologic, neurologic, hepatologic, rheumatologic, cardiologic, and dermatologic diseases. The frequency of comorbidity with autoimmune disorders is estimated to be 10 times higher than that in the normal population [4]. In our study, 10 CD patients (19.2%) had a coexisting disease. Vaccination is an efficient and reliable way to protect the population from common HBV infections [4]. In a study perfomed in eastern Turkey, the seroprevalance of HBV was found to be 9.4%. Among 1091 school children, only 32 had a history of HBV immunization, and the overall HBV vaccination rate was very low, at only 2.9%. Mothers’ educational status, socio-economic status, and number of siblings were 3 important factors that contributed to the protection of children from HBV in Turkey [16]. Furthermore, old age, smoking, obesity, and male gender are factors that may predispose an individual to be a nonresponder to HBV vaccination [4][8]. In our study, evaluation of anti-HBs titers revealed that anti-HBs negativity was higher in male CD patients than in female CD patients (P = 0.0001). A significantly higher incidence of nonresponsiveness to HBV vaccination has been shown to be related to MHC and HLA-II antigens and homozygotes for the alleles HLA-B8, DR3, and DQ2 [4][5]. CD is also known to be associated with HLA-II, specifically HLA-DQ2 and HLA-DQ8, and it has been demonstrated that HLA-II antigens play a role in nonresponsiveness to the hepatitis B vaccine [4]. It is thought that both HBsAg protein fragments and gliadin peptides bind to HLA-DQ2 molecules, and their competition for HLA-DQ2 binding may result in a defective antibody response against the recombinant HBsAg vaccine in active CD [5][8]. In our study, the response rate of CD patients to HBV vaccination was found to be significantly lower than that of patients in the control group (P < 0.05). Park et al. [6] demonsrated that 53.9% of children with CD did not show a response to standard vaccination regimens for HBV; however, the response to other childhood vaccines was not impaired, supporting the hypothesis that HLA haplotypes may play a role in responding to the HBV vaccine. Nemes et al. [8] administered the HBV vaccine to 22 prospective CD patients just after diagnosis during dietary treatment and found that seroconversion after HBV vaccination was 95.5%. All of these patients carried HLA-DQ2. They then further demonstrated that HLA-DQ alleles did not appear to have a primary role in nonresponsiveness to the HBV vaccine. Ahishali et al. (4) found that 68% of adults with CD were responsive to HBV vaccination; this percentage was significantly lower than that in the general population (90–95%). They also found that 50% of the apatients who had additional autoimmune disease along with CD were nonresponders to the HBV vaccine, emphasizing the involvement of genetic factors in the pathogenesis. In our study, although anti-HBs negativity was higher in CD patients who had an additional coexisting disease than in patients who did not have a coexisting disease, this difference was not statistically significant. In addition, the 7 CD patients who also had autoimmune hepatitis or insulin-dependent diabetes mellitus could have been nonresponsive to HBV vaccination because of other diseases unrelated to CD. Ertem et al. [11] evaluated anti-HBs titers in CD patients and healthy children. They found that anti-HBs negativity was significantly higher in CD patients than in healthy controls. They also demonstrated that response to the HBV vaccine in children with CD who were compliant with the GFD was not different from that in the healthy population. In our study, anti-HBs positivity was significantly higher in celiac patients who were compliant to GFD than in those who were noncompliant (P < 0.05). These results suggest that there may be an important relationship between anti-HBs titers and compliance with the GFD. Nonresponsiveness of celiac patients to the HBV vaccine may represent a significant public health problem. Infact, there may be a large population of HBV-susceptible individuals since there is a high frequency of CD worldwide [5]. In our country, where the prevalence of CD is high, nonresponsiveness to the HBV vaccine in CD patients may hinder the immunization of populations by national HBV vaccination. Nonresponsiveness to the HBV vaccine was higher in children with CD than in normal healthy children. Therefore, response to the HBV vaccine should be investigated in children with CD, and a different immunization schedule may need to be developed. Furthermore, nonresponsiveness to the HBV vaccine may be considered a sign of a possible undiagnosed celiac disease. Further studies are required to enhance the HBV vaccine response in CD patients.
